# The Case of Sir Renjamin Brodie

**Published:** 1860-11

**Authors:** 


THE CASE OF SIR BENJAMIN BRODIE.—The medical profession, both in England and abroad, have recently evinced the liveliest interest in the distressing case of this distinguished gentleman, who, at the advanced age of eighty-four, has undergone the operation of iridectomy for impaired vision in both eyes. About last December his sight began to fail, and on the twelfth of July of this year Mr. Bowman removed a portion of each iris for supposed glaucoma. There seems to have been an error in the diagnosis, Mr. Hodgson and Mr. White Cooper regarding the disease as senile cataract, and did not recommend the operation of iridectomy. The left eye is very little improved, if at all, and in the right vision is completely lost; but as there is now a cataract very evident in the latter, sight may be restored by extraction of the lens, provided the eye is not glaucomatous. The high position of the unfortunate and much respected sufferer renders the case especially interesting in relation to the operation of iridectomy, one of equivocal value, which is now being so extensively tried.

